# Supplementary material for: Circadian rhythm profiles derived from accelerometer measures of the sleep-wake cycle in two cohort studies
Source: Nat Commun. 2025 Dec 13;16:11357. doi: 10.1038/s41467-025-66407-2 (PMC12727707; doi:10.1038/s41467-025-66407-2)
Supplement: Supplementary file 1 — Supplementary Information [file 41467_2025_66407_MOESM1_ESM.pdf]

SUPPLEMENTARY MATERIAL

Circadian rhythm profiles derived from accelerometer measures of the sleep-wake cycle in two cohort studies

Sam Vidil, PhD candidate [1]; Ian Meneghel Danilevicz, PhD[1]; Aline Dugravot, MSc[1]; Aurore Fayosse, MSc[1]; Benjamin Landré, PhD[1]; Vincent van Hees, PhD[2]; Mathilde Chen, PhD[3,4]; Archana Singh-Manoux, PhD[1,5]; Séverine Sabia, PhD[1,5*]

[1]Université Paris Cité, Inserm U1153, Center for Research in Epidemiology and Statistics (CRESS), Epidemiology of Ageing and Neurodegenerative diseases (EpiAgeing), Paris, France

[2]Accelting, Almere, The Netherlands

[3]CIRAD, UMR PHIM, f-34398 Montpellier, France

[4]PHIM, Univ Montpellier, CIRAD, INRAE, Institut Agro, IRD, 34398 Montpellier, France

[5]Faculty of Brain Sciences, University College London, UK

*Address for correspondence

Faculty of Brain Sciences, University College London, UK

Email: s.sabia@ucl.ac.uk

SUPPLEMENTARY TABLES

Table S1. Definition of the metrics of circadian rhythm dimensions.

Table S2. Principal component analyses: selection of the retained principal components for the metrics used in the literature.

Table S3. Factors loadings of the three principal components retained in the analysis with the metrics used in the literature in the Whitehall II and UK Biobank accelerometer sub-studies.

Table S4. Principal component analyses: selection of the retained principal components.

Table S5. Factors loadings of the eight principal components retained in the analysis in the Whitehall II accelerometer sub-study.

Table S6. Factors loadings of the eight principal components retained in the analysis in the UK Biobank accelerometer sub-study.

Table S7. Unstandardized mean (standard deviation) scores and ranking of each metric across the circadian rhythm clusters in the Whitehall II accelerometer sub-study.

Table S8. Unstandardized mean (standard deviation) scores and ranking of each metric across the circadian rhythm clusters in the UK Biobank accelerometer sub-study.

Table S9. Subjective chronotype preference prevalence in the identified circadian rhythm clusters in the UK Biobank accelerometer sub-study.

Table S10. Distribution of shift work in the identified clusters in the UK Biobank accelerometer sub-study.

SUPPLEMENTARY FIGURES

Figure S1. Flow-chart of sample selection in the Whitehall II accelerometer sub-study.

Figure S2. Flow-chart of sample selection in the UK Biobank accelerometer sub-study.

Figure S3. Work-flow of the statistical analysis for clusters identification.

Figure S4. Statistical criteria results for the clustering in Whitehall II (n=3,991).

Figure S5. Statistical criteria results for the clustering in UK Biobank (n=54.995).

Figure S6. Illustration of acceleration signals for typical profiles for the nine clusters identified in the Whitehall II accelerometer sub-study.

Figure S7. Illustration of acceleration signals for typical profiles for the nine clusters identified in the UK Biobank accelerometer sub-study.

Table S1. Definition of the metrics of circadian rhythm dimensions.

Metrics	Name in GGIR	Description	Period of observation
**Dimension: Rest-activity rhythm (RAR)**			
Relative amplitude	(m10value - l5value) / (m10value + l5value)	Calculated as $\frac{M_{10}-L_5}{M_{10}+L_5}$ with M_{10} corresponding to the mean acceleration of the most active 10-hour period and L_5 the mean acceleration of the least active 5-hour period. Higher values indicate larger amplitude in the rhythm.	Calculated for each full day (waking and sleeping periods) and averaged over valid days.
Cosinor mesor	cosinor_mes	The mean value of the cosinor function fitted to the log transformed acceleration signal (ENMO time series). Higher values indicate more activity.	Calculated using the full observation period (including day and night periods), non-wear periods were omitted.
Cosinor amplitude	cosinor_amp	The amplitude of the cosinor function fitted to the log transformed acceleration signal, corresponding to the peak of the function minus the mesor. Higher values indicate larger amplitude in the rhythm.	
Cosinor R^2	cosinor_r2	Measure of goodness of fit of the cosinor function to the log transformed acceleration signal. Higher values indicate better goodness of fit.	
Interdaily stability (IS)	is	Calculated as $\mathrm{IS} = \frac{P\sum_{h=1}^{H}(\bar{x}_h-\bar{x})^2}{H\sum_{p=1}^{P}(x_p-\bar{x})^2}$, where *H* is the number of hours per day, *P* the total number of hours over the observation period, x_p is the pth element of a vector of P hourly proportions of activity , $\bar{x}_h$ is the hth element of a vector of *H* hourly proportions of being active (defined as mean acceleration >40 m*g*), and $\bar{x}$ is the overall mean hourly proportion of being active over the entire period. IS measures how constant is the routine of activity over several days and ranges from 0 to 1, values close to 1 indicate more constant routine.	
Intradaily variability (IV)	iv	Calculated as $\mathrm{IV} = \frac{P\sum_{p=2}^{P}(x_p-x_{p-1})^2}{(P-1)\sum_{p=1}^{P}(x_p-\bar{x})^2}$ See notations in the row above. It measures the variability in activity hour by hour throughout the days. It ranges from 0 to +∞, value close to 2 indicates more fragmented rhythm, and >2 indicates ultradian rhythm (very uncommon).	

Table S1 continued.

Metrics	Name in GGIR	Description	Period of observation
**Dimension: Daytime activity**			
SB, LIPA, and MVPA durations	SB: dur_day_total_in_min_pla<br>LIPA: dur_day_total_lig_min_pla<br>MVPA: dur_day_total_mod_min_pla + dur_day_total_vig_min_pla	Total daily time during waking period spent in 60s-epoch acceleration<40 m*g* for SB (in hours), 40-99 m*g* for LIPA (in hours), ≥100 m*g* for MVPA (in minutes)	Calculated for each waking period and averaged over valid days. Non-wear periods of valid days were imputed using the average of the signal at the same time of the day on other valid days.
Number of SB, LIPA, and MVPA bouts	SB: frag_nfrag_in_day_pla<br>LIPA: frag_nfrag_lipa_day_pla<br>MVPA: frag_nfrag_mvpa_day_pla	Number of bouts spent in SB, LIPA, and MVPA per day. Higher numbers denote more fragmented episodes in the activity level.	
Mean duration of SB, LIPA, and MVPA bouts	SB: dur_day_total_in_min_pla / data_acc2$frag_nfrag_in_day_pla<br>LIPA: dur_day_total_lig_min_pla / frag_nfrag_lipa_day_pla<br>MVPA: (dur_day_total_mod_min_pla + dur_day_total_vig_min_pla) / frag_nfrag_mvpa_day_pla	Average duration of bouts (in minutes) in SB, LIPA, and MVPA, computed as the total daily duration during waking period divided by daily number of bouts. Longer mean durations in an activity level represent less fragmented activity.	
Intensity gradient intercept and slope	ig_day_intercept_pla<br><br>ig_day_gradient_pla	Intensity gradient (IG) relies on the acceleration distribution during the waking period per day defined as the time spent (ordinate) in each acceleration (abscissa) over the day. IG intercept and slope are extracted from the linear regression between log transformed abscissa and log transformed ordinate.<br>Examples: Higher intercept and steeper gradient (i.e. more negative gradient) represent more time in SB and little time spent in midrange and higher intensity. Lower intercept and shallow gradient correspond to more time spread across the range of intensities.	
Acceleration during waking	acc_day_mg_pla	Mean acceleration during waking period (in m*g*). Higher values represent higher activity level over the day.	
M_{10} mean acceleration	m10value	Mean acceleration during the most active 10-hour period (in m*g*) of the entire day. Higher values represent higher activity level over the most active hours.	Calculated for each full day window. Non-wear periods of valid days were imputed using the average of the signal at the same time of the day on other valid days.
Transition probability from activity to rest during the day ($TP_{ar,d}$)	frag_tp_pa2in_day	Calculated as $\mathrm{TP}_{\mathrm{ar,d}} = \frac{n_{a,d}+\delta}{T_{a,d}+\delta}$, where $n_{a,d}$ is the number of bouts of activity during the day (waking period), $T_{a,d}$ is the total time of activity during the day (waking period), and δ is a small number, 10^{-6}, to avoid potential division by zero.<br>It corresponds to the probability of transitioning from a physically active (LIPA or MVPA) to sedentary state and ranges from 0 to 1, values close to 1 indicate frequent switching from one state to the other.	Calculated using the full observation period (including only valid day periods), sleep and non-wear periods were omitted.
Transition probability rest to activity during the day ($TP_{ra,d}$)	frag_tp_in2pa_day	Calculated as $\mathrm{TP}_{\mathrm{ra,d}} = \frac{n_{r,d}+\delta}{T_{r,d}+\delta}$, where δ is 10^{-6}, $n_{r,d}$ the number of bouts of rest during the day (waking period), and $T_{r,d}$ the total time of rest during the day (waking period).<br>It corresponds to the probability of transitioning from a sedentary to physically active (LIPA or MVPA) state and ranges from 0 to 1.	

Table S1. continued

Metrics	Name in GGIR	Description	Period of observation
**Dimension: Sleep**			
Sleep duration	dur_spt_sleep_min_pla	Duration (in hours) of time spent sleeping during the sleep period.	Calculated for each sleep period and averaged over valid days. Non-wear periods of valid days were imputed using the average of the signal at the same time of the day on other valid days.
Sleep efficiency	sleep_efficiency_pla	Percent of time spent sleeping during the sleep period. Higher values denote a better sleep quality.	
Mean duration of sleep bouts	dur_spt_sleep_min_pla / nblocks_spt_sleep_pla	Mean duration (in minutes) of sleep bouts during the sleep period. Longer mean duration denotes longer sleep bouts.	
Number of sleep bouts	nblocks_spt_sleep_pla	Number of sleep bouts during the sleep period. More sleep bouts denote more fragmented sleep.	
Mean acceleration during sleep	acc_spt_sleep_mg_pla	Mean acceleration during sleep (in m*g*). For cluster calculation, this variable was log transformed to reduce its skewness. Higher values denote more movements during the sleep.	
Duration of wake after sleep onset (WASO)	dur_spt_min_pla - dur_spt_sleep_min_pla	Time spent awake (in minutes) during the sleep period. Higher values indicate more fragmented sleep.	
Mean duration of wake bouts	(dur_spt_min_pla - dur_spt_sleep_min_pla) / (nblocks_spt_sleep_pla - 1)	Mean duration of bouts spent awake during sleep period (in minutes). Longer duration denotes more difficulties falling asleep when awake.	
L_5 mean acceleration	l5value	Mean acceleration during the 5 least active hours (in m*g*). Higher values indicate more activity during the 5 least active hours (commonly during the sleep period).	Calculated for each full day window. Non-wear periods of valid days were imputed using the average of the signal at the same time of the day on other valid days.
Transition probabilities from wake to sleep during the night ($TP_{ws,n}$)	frag_tp_wake2sleep_spt	Calculated as $TP_{ws,n} = \frac{n_{w,n}+\delta}{T_{w,n}+\delta}$, where δ is 10^{-6}, $n_{w,n}$ the number of bouts of wake during the night, and $T_{w,n}$ the total waking time during the night. It corresponds to the probability of transitioning from wake to sleep state during the sleep period and range from 0 to 1, value close to 1 indicates frequent changes between wake to sleep states.	Calculated using the full observation period (including only sleep periods), waking and non-wear periods were omitted.
TP from sleep to wake during sleep period ($TP_{sw,n}$)	frag_tp_sleep2wake_spt	Probability of transitioning from sleep to wake state during the sleep period Range from 0 to 1. Value close to 1 indicates frequent changes from sleep to wake states.	
**Dimension: Chronotype**			
Sleep onset	sleeponset_pla	Timing of the sleep onset (in hours of the day)	Calculated for each sleep period and averaged over valid days.
Waking time	wakeup_pla - 24	Timing of the waking up to start the day (in hours of the day)	
M_{10} start	m10time_num	Timing of the start of M_{10} (in hours of the day)	Calculated for each entire day (waking and sleeping periods) and averaged over valid days.
L_5 start	l5time_num	Timing of the start of L_5 (in hours of the day)	
Cosinor acrotime	cosinor_acrotime	Time at which the cosinor function fitted to the log transformed acceleration signal reaches its maximum	Calculated using the full observation period (including day and night periods), non-wear periods were omitted.

Abbreviations: IG, intensity gradient; L_5, least 5 active hours; LIPA, light intensity physical activity; M_{10}, most active 10-hour period; MVPA, moderate to vigorous physical activity; SB, sedentary behaviour; $TP_{ar,d}$, transition probability from activity to rest during the day; $TP_{ra,d}$, transition probability from rest to activity during the day; $TP_{sw,n}$, transition probability from sleep to wake during the night; $TP_{ws,n}$, transition probability from wake to sleep during the night; WASO, wake after sleep onset.
Definitions: A *bout* corresponds to an uninterrupted episode spent in a given range of state; *Sleep period* is from sleep onset to waking time to start the day.
Remark: Cosinor analysis, IS, and IV invalid data points are set to missing, while for all other analysis invalid timestamps were imputed by average of valid values at the same time on other days of the recording as previously described [7].

Table S2. Principal component analyses: selection of the retained principal components for the metrics used in the literature.

	Eigenvalue	Variance explained	Cumulative variance explained
Whitehall II			
Component 1	4.03	40.3	40.3
Component 2	2.41	24.1	64.4
Component 3	1.60	16.0	80.4
Component 4	0.61	6.1	86.5
Component 5	0.50	5.0	91.5
Component 6	0.39	3.9	95.4
Component 7	0.19	1.9	97.3
Component 8	0.12	1.2	98.5
Component 9	0.09	0.9	99.4
Component 10	0.06	0.6	100.0
UK Biobank			
Component 1	3.71	37.1	37.1
Component 2	2.25	22.5	59.6
Component 3	1.80	18.0	77.6
Component 4	0.66	6.6	84.2
Component 5	0.51	5.1	89.2
Component 6	0.43	4.3	93.5
Component 7	0.25	2.5	96.0
Component 8	0.20	2.0	98.1
Component 9	0.14	1.4	99.4
Component 10	0.06	0.6	100.0

Last component retained is highlighted in yellow, corresponding to the last component with an eigenvalue ≥1 and a cumulative variance explained ≥75%.

Table S3. Factors loadings of the three principal components retained in the analysis with the metrics used in the literature in the Whitehall II and UK Biobank accelerometer sub-studies.

	PC1	PC2	PC3
Whitehall II			
Relative amplitude	0.41	-0.10	-0.36
Cosinor mesor	0.35	-0.14	0.46
Cosinor amplitude	0.45	-0.11	-0.15
Interdaily stability (IS)	0.36	0.07	0.03
Intradaily variability (IV)	-0.38	0.06	-0.04
M_{10} mean acceleration	0.43	-0.14	0.19
L_5 mean acceleration	-0.08	-0.02	0.76
M_{10} start	-0.15	-0.55	0.00
L_5 start	-0.09	-0.52	-0.11
Cosinor acrotime	-0.11	-0.60	0.00
UK Biobank			
Relative amplitude	0.37	-0.18	-0.43
Cosinor mesor	0.31	-0.07	0.49
Cosinor amplitude	0.46	-0.16	-0.10
Interdaily stability (IS)	0.37	0.03	0.03
Intradaily variability (IV)	-0.39	0.09	-0.12
M_{10} mean acceleration	0.43	-0.10	0.26
L_5 mean acceleration	-0.13	0.14	0.69
M_{10} start	-0.16	-0.55	0.07
L_5 start	-0.14	-0.51	0.07
Cosinor acrotime	-0.15	-0.59	0.08

Abbreviations: IG, intensity gradient; L_5, least 5 active hours; LIPA, light intensity physical activity; M_{10}, most active 10-hour period; MVPA, moderate to vigorous physical activity; PC, principal component; SB, sedentary behaviour; $TP_{ar,d}$, transition probability from activity to rest during the day; $TP_{ra,d}$, transition probability from rest to activity during the day; $TP_{sw,n}$, transition probability from sleep to wake during the night; $TP_{ws,n}$, transition probability from wake to sleep during the night; WASO, wake after sleep onset.

**Colour legend**:	0.10 ≤ |coef| < 0.15	0.15 ≤ |coef| < 0.20	0.20 ≤ |coef| < 0.25	0.25 ≤ |coef| < 0.30	|coef| ≥ 0.30

Table S4. Principal component analyses: selection of the retained principal components.

	Eigenvalue	Variance explained	Cumulative variance explained
Whitehall II			
Component 1	12.31	34.2	34.2
Component 2	5.77	16.0	50.2
Component 3	3.74	10.4	60.6
Component 4	2.86	7.9	68.5
Component 5	2.60	7.2	75.8
Component 6	1.52	4.2	80.0
Component 7	1.24	3.4	83.5
Component 8	1.09	3.0	86.5
Component 9	0.82	2.3	88.8
Component 10	0.74	2.1	90.8
UK Biobank			
Component 1	11.04	30.7	30.7
Component 2	5.10	14.2	44.8
Component 3	3.69	10.2	55.1
Component 4	3.28	9.1	64.2
Component 5	2.91	8.1	72.3
Component 6	1.76	4.9	77.1
Component 7	1.56	4.3	81.5
Component 8	1.40	3.9	85.4
Component 9	0.86	2.4	87.8
Component 10	0.68	1.9	89.6

Last component retained is highlighted in yellow, corresponding to the last component with an eigenvalue ≥1 and a cumulative variance explained ≥75%.

Table S5. Factors loadings of the eight principal components retained in the analysis in the Whitehall II accelerometer sub-study.

	PC1	PC2	PC3	PC4	PC5	PC6	PC7	PC8
Rest-activity rhythm								
Relative amplitude	-0.22	0.13	0.00	0.15	-0.22	-0.18	0.03	-0.03
Cosinor mesor	-0.23	-0.11	-0.04	-0.14	0.19	-0.01	0.03	0.01
Cosinor amplitude	-0.24	0.06	0.01	0.02	-0.23	-0.09	-0.05	0.03
Cosinor R^2	-0.20	0.06	0.03	-0.05	-0.32	-0.05	-0.07	0.08
Interdaily stability (IS)	-0.17	0.01	0.10	-0.07	-0.13	0.13	-0.09	-0.01
Intradaily variability (IV)	0.19	0.00	-0.03	0.01	0.12	-0.07	0.04	0.00
Daytime activity								
SB duration	0.25	0.02	0.03	-0.01	0.11	-0.26	0.14	-0.07
Number of SB bouts	-0.19	-0.05	-0.04	-0.32	0.00	-0.13	0.26	0.26
Mean duration of SB bouts	0.22	0.03	0.02	0.17	0.03	-0.01	-0.15	-0.21
$TP_{ar,d}$	0.23	0.01	0.00	-0.14	-0.08	-0.06	0.21	0.36
LIPA duration	-0.24	-0.05	-0.01	-0.26	0.00	0.01	-0.05	-0.15
MVPA duration	-0.23	-0.01	-0.03	0.24	0.17	-0.08	-0.01	-0.05
Number of LIPA bouts	-0.25	-0.05	-0.04	-0.22	0.04	-0.10	0.15	0.10
Number of MVPA bouts	-0.24	-0.03	-0.04	0.07	0.11	-0.04	-0.02	-0.12
Mean duration of LIPA bouts	-0.14	-0.03	0.04	-0.19	-0.08	0.17	-0.35	-0.50
Mean duration of MVPA bouts	-0.07	0.04	0.01	0.40	0.19	-0.13	0.03	0.15
$TP_{ra,d}$	-0.24	-0.05	-0.04	-0.23	-0.04	0.02	0.10	0.19
M_{10} mean acceleration	-0.26	-0.02	-0.02	0.13	0.10	-0.05	-0.04	-0.04
Acceleration during waking	-0.27	-0.03	-0.04	0.09	0.10	-0.01	-0.02	0.01
IG intercept	0.17	-0.02	0.03	-0.37	-0.14	-0.02	0.00	-0.15
IG slope	-0.21	0.01	-0.03	0.30	0.15	-0.01	0.02	0.09
Sleep								
Sleep duration	-0.01	0.27	-0.03	0.10	-0.22	0.45	-0.10	0.26
Sleep efficiency	-0.03	0.40	-0.02	-0.06	0.08	0.06	0.07	-0.03
Mean duration of sleep bouts	0.01	0.33	-0.02	-0.10	0.13	-0.04	-0.31	0.19
$TP_{ws,n}$	-0.04	0.32	-0.02	0.01	0.04	0.16	0.38	-0.21
Mean acceleration during sleep	0.00	-0.09	-0.01	-0.08	0.38	0.51	0.01	0.10
Number of sleep bouts	-0.01	-0.29	0.00	0.18	-0.26	0.27	0.29	-0.07
L_5 mean acceleration	0.00	-0.21	-0.03	-0.15	0.43	0.23	-0.02	0.03
$TP_{sw,n}$	0.00	-0.36	0.01	0.11	-0.13	0.05	0.27	-0.16
WASO	0.03	-0.38	0.02	0.10	-0.16	0.06	-0.11	0.11
Mean duration of wake bouts	0.05	-0.30	0.02	-0.03	0.01	-0.20	-0.46	0.24
Chronotype								
Sleep onset	0.04	-0.02	-0.42	-0.07	0.13	-0.24	0.06	-0.25
Waking time	0.04	0.01	-0.43	0.08	-0.18	0.23	-0.11	0.07
M_{10} start	0.04	-0.03	-0.43	0.02	-0.02	0.04	-0.06	0.09
L_5 start	0.02	0.01	-0.45	0.02	-0.07	0.02	0.00	-0.09
Cosinor acrotime	0.01	-0.02	-0.47	-0.04	-0.03	-0.05	0.01	0.07

Abbreviations: IG, intensity gradient; L_5, least 5 active hours; LIPA, light intensity physical activity; M_{10}, most active 10-hour period; MVPA, moderate to vigorous physical activity; PC, principal component; SB, sedentary behaviour; $TP_{ar,d}$, transition probability from activity to rest during the day; $TP_{ra,d}$, transition probability from rest to activity during the day; $TP_{sw,n}$, transition probability from sleep to wake during the night; $TP_{ws,n}$, transition probability from wake to sleep during the night; WASO, wake after sleep onset.

Colour legend: 0.10 ≤ |coef| < 0.15 | 0.15 ≤ |coef| < 0.20 | 0.20 ≤ |coef| < 0.25 | 0.25 ≤ |coef| < 0.30 | |coef| ≥ 0.30

Table S6. Factors loadings of the eight principal components retained in the analysis in the UK Biobank accelerometer sub-study.

	PC1	PC2	PC3	PC4	PC5	PC6	PC7	PC8
Rest-activity rhythm								
Relative amplitude	0.18	-0.13	-0.21	-0.11	0.27	0.12	-0.09	-0.04
Cosinor mesor	0.24	0.09	0.20	0.06	-0.15	0.01	-0.05	0.02
Cosinor amplitude	0.24	-0.05	-0.08	-0.05	0.22	0.12	0.00	0.11
Cosinor R^2	0.20	-0.05	-0.05	-0.03	0.30	0.11	0.00	0.12
Interdaily stability (IS)	0.18	-0.01	-0.04	0.07	0.13	-0.01	0.14	0.20
Intradaily variability (IV)	-0.20	-0.02	0.00	-0.01	-0.10	-0.01	-0.06	-0.11
Daytime activity								
SB duration	-0.27	0.03	-0.01	0.05	-0.05	0.19	-0.20	0.09
Number of SB bouts	0.13	0.04	0.33	0.12	0.12	0.02	-0.23	-0.31
Mean duration of SB bouts	-0.22	-0.01	-0.19	-0.04	-0.10	0.09	0.04	0.23
$TP_{ar,d}$	-0.23	-0.01	0.08	0.04	0.10	0.00	-0.20	-0.32
LIPA duration	0.21	0.02	0.29	0.11	0.11	-0.03	0.12	0.14
MVPA duration	0.25	0.04	-0.11	-0.07	-0.21	0.11	-0.09	0.03
Number of LIPA bouts	0.24	0.05	0.24	0.07	0.03	0.05	-0.15	-0.12
Number of MVPA bouts	0.26	0.04	0.02	-0.03	-0.12	0.08	-0.08	0.03
Mean duration of LIPA bouts	0.05	-0.03	0.17	0.10	0.16	-0.13	0.42	0.44
Mean duration of MVPA bouts	0.12	0.02	-0.28	-0.11	-0.26	0.09	-0.07	-0.01
$TP_{ra,d}$	0.23	0.03	0.24	0.06	0.08	-0.08	-0.03	-0.23
M_{10} mean acceleration	0.27	0.04	-0.07	-0.05	-0.15	0.09	-0.03	0.06
Acceleration during waking	0.28	0.03	-0.04	-0.05	-0.14	0.03	-0.01	-0.03
IG intercept	-0.17	0.00	0.26	0.14	0.22	0.03	-0.03	0.12
IG slope	0.22	0.02	-0.20	-0.12	-0.21	0.00	0.00	-0.08
Sleep								
Sleep duration	0.06	-0.23	-0.15	-0.12	0.11	-0.37	0.27	-0.31
Sleep efficiency	0.03	-0.42	0.05	0.03	-0.09	-0.06	-0.03	0.00
Mean duration of sleep bouts	0.00	-0.35	0.05	0.05	-0.08	0.21	0.26	-0.18
$TP_{ws,n}$	0.04	-0.28	0.02	-0.03	-0.05	-0.35	-0.37	0.23
Mean acceleration during sleep	-0.01	0.08	0.16	0.07	-0.38	-0.32	0.22	-0.08
Number of sleep bouts	0.02	0.31	-0.14	-0.13	0.17	-0.38	-0.08	-0.01
L_5 mean acceleration	-0.01	0.17	0.23	0.10	-0.39	-0.13	0.12	0.08
$TP_{sw,n}$	-0.01	0.40	-0.06	-0.06	0.09	-0.15	-0.18	0.13
WASO	-0.01	0.39	-0.11	-0.08	0.14	-0.08	0.13	-0.12
Mean duration of wake bouts	-0.04	0.26	0.00	0.04	0.03	0.40	0.38	-0.22
Chronotype								
Sleep onset	-0.07	0.04	0.26	-0.31	-0.12	0.22	-0.11	0.26
Waking time	-0.02	-0.07	0.07	-0.45	0.04	-0.17	0.21	-0.09
M_{10} start	-0.04	-0.01	0.17	-0.40	0.01	0.04	0.02	-0.05
L_5 start	-0.04	-0.01	0.17	-0.41	-0.01	0.05	0.04	0.09
Cosinor acrotime	-0.03	-0.01	0.23	-0.43	0.03	0.07	-0.03	-0.07

Abbreviations: IG, intensity gradient; L_5, least active 5-hour period; LIPA, light intensity physical activity; M_{10}, most active 10-hour period; MVPA, moderate to vigorous physical activity; PC, principal component; SB, sedentary behaviour; $TP_{ar,d}$, transition probability from activity to rest during the day; $TP_{ra,d}$, transition probability from rest to activity during the day; $TP_{sw,n}$, transition probability from sleep to wake during the night; $TP_{ws,n}$, transition probability from wake to sleep during the night; WASO, wake after sleep onset.

Colour legend: 0.10 ≤ |coef| < 0.15 | 0.15 ≤ |coef| < 0.20 | 0.20 ≤ |coef| < 0.25 | 0.25 ≤ |coef| < 0.30 | |coef| ≥ 0.30

Table S7. Unstandardized mean (standard deviation) scores and ranking* of each metric across the circadian rhythm clusters in the Whitehall II accelerometer sub-study.

	Total study population	Cluster 1	Cluster 2	Cluster 3	Cluster 4	Cluster 5	Cluster 6	Cluster 7	Cluster 8	Cluster 9
		RAR ++ PA ++	RAR + LIPA + Sleep -	RAR + LIPA + Sleep +	MVPA ++	RAR - Chronotype --	RAR - PA - Sleep +	RAR - PA - Sleep --	RAR - PA + Restless sleep	RAR -- PA --
N (%)	3991	501 (12.6)	587 (14.7)	626 (15.7)	379 (9.5)	333 (8.3)	705 (17.7)	366 (9.2)	234 (5.9)	260 (6.5)
Rest-activity rhythm										
Relative amplitude	0.78 (0.08)	0.86 (0.03)[h]	0.80 (0.03)[e]	0.82 (0.04)[f]	0.84 (0.04)[g]	0.76 (0.06)[d]	0.75 (0.05)[c]	0.69 (0.07)[b]	0.74 (0.07)[c]	0.63 (0.08)[a]
Cosinor mesor	2.47 (0.21)	2.72 (0.12)[f]	2.50 (0.11)[e]	2.50 (0.11)[e]	2.50 (0.12)[e]	2.45 (0.15)[d]	2.30 (0.12)[b]	2.38 (0.14)[c]	2.78 (0.14)[g]	2.15 (0.15)[a]
Cosinor amplitude	0.84 (0.20)	1.12 (0.12)[g]	0.92 (0.11)[f]	0.93 (0.11)[f]	0.92 (0.14)[f]	0.76 (0.12)[d]	0.72 (0.10)[c]	0.65 (0.12)[b]	0.83 (0.15)[e]	0.51 (0.13)[a]
Cosinor R^2	0.28 (0.09)	0.38 (0.07)[g]	0.32 (0.07)[f]	0.32 (0.07)[f]	0.28 (0.07)[e]	0.24 (0.07)[c]	0.23 (0.06)[c]	0.20 (0.06)[b]	0.26 (0.08)[d]	0.16 (0.07)[a]
Interdaily stability (IS)	0.53 (0.12)	0.64 (0.09)[f]	0.56 (0.09)[e]	0.57 (0.09)[e]	0.53 (0.10)[d]	0.46 (0.10)[b]	0.49 (0.10)[c]	0.46 (0.10)[b]	0.57 (0.11)[e]	0.38 (0.11)[a]
Intradaily variability (IV)	0.99 (0.25)	0.74 (0.16)[a]	0.90 (0.18)[b]	0.93 (0.18)[b]	0.94 (0.21)[b]	1.07 (0.20)[c]	1.11 (0.21)[d]	1.14 (0.22)[d]	0.90 (0.20)[b]	1.37 (0.26)[e]
Daytime activity										
SB duration (hours)	12.0 (1.7)	9.6 (0.9)[a]	11.5 (0.9)[c]	11.6 (0.8)[c]	11.6 (1.0)[c]	12.5 (1.1)[d]	13.2 (0.8)[e]	13.3 (1.3)[e]	10.9 (1.3)[b]	14.7 (1.2)[f]
Number of SB bouts	71.0 (16.0)	82.5 (12.4)[f]	78.5 (12.1)[e]	78.8 (10.9)[e]	65.7 (8.9)[c]	72.0 (13.9)[d]	61.7 (10.1)[b]	63.9 (12.2)[c]	84.9 (13.3)[f]	42.0 (12.3)[a]
Mean duration of SB bouts (min)	11.1 (5.2)	7.1 (1.2)[a]	9.0 (1.6)[c]	9.0 (1.4)[c]	10.8 (1.9)[d]	10.9 (2.4)[d]	13.2 (2.6)[e]	13.1 (4.1)[e]	7.9 (1.6)[b]	23.6 (10.6)[f]
$TP_{ar,d}$ (%)	28.9 (7.8)	20.4 (3.6)[a]	28.1 (4.7)[c]	27.5 (4.5)[c]	23.0 (3.9)[b]	30.1 (5.6)[d]	33.3 (5.6)[e]	33.6 (5.9)[e]	23.5 (4.5)[b]	43.2 (8.6)[f]
LIPA duration (hours)	3.5 (1.2)	5.0 (0.8)[f]	3.9 (0.6)[d]	3.9 (0.6)[d]	3.2 (0.6)[c]	3.3 (0.7)[c]	2.6 (0.5)[b]	2.7 (0.7)[b]	4.8 (1.0)[e]	1.5 (0.5)[a]
MVPA duration (min)	56.0 (38.4)	108.6 (37.1)[g]	48.1 (19.1)[c]	54.1 (21.0)[d]	97.3 (27.9)[f]	46.9 (25.1)[c]	30.0 (16.0)[b]	30.2 (17.5)[b]	83.8 (38.5)[e]	10.0 (8.7)[a]
Number of LIPA bouts	85.8 (21.6)	112.5 (12.6)[g]	93.3 (11.8)[e]	94.3 (11.2)[e]	83.7 (10.9)[d]	84.2 (15.4)[d]	69.5 (10.4)[b]	72.1 (13.5)[c]	108.7 (15.4)[f]	44.7 (13.1)[a]
Number of MVPA bouts	23.2 (13.5)	43.2 (11.6)[g]	23.1 (8.1)[d]	24.2 (8.1)[d]	29.6 (9.6)[e]	20.8 (9.2)[c]	13.4 (5.9)[b]	13.7 (6.7)[b]	35.1 (13.0)[f]	5.1 (3.7)[a]
Mean duration of LIPA bouts (min)	2.4 (0.4)	2.7 (0.4)[f]	2.5 (0.3)[d]	2.5 (0.3)[d]	2.3 (0.3)[bc]	2.3 (0.3)[c]	2.3 (0.3)[b]	2.3 (0.3)[b]	2.6 (0.4)[e]	2.0 (0.3)[a]
Mean duration of MVPA bouts (min)	2.3 (0.8)	2.5 (0.6)[e]	2.1 (0.5)[b]	2.2 (0.5)[cd]	3.5 (1.2)[f]	2.2 (0.7)[bc]	2.2 (0.8)[bc]	2.1 (0.7)[b]	2.4 (0.6)[d]	1.8 (0.9)[a]
$TP_{ra,d}$ (%)	10.2 (3.2)	14.5 (2.6)[f]	11.5 (2.0)[d]	11.4 (1.8)[d]	9.5 (1.5)[c]	9.7 (2.2)[c]	7.8 (1.4)[b]	8.1 (1.8)[b]	13.2 (2.7)[e]	4.8 (1.5)[a]
M_{10} mean acceleration (m*g*)	39.5 (12.5)	58.1 (10.0)[g]	39.5 (4.6)[d]	40.7 (5.2)[e]	50.3 (10.9)[f]	35.9 (6.4)[c]	29.7 (3.9)[b]	30.1 (5.1)[b]	49.2 (8.5)[f]	20.2 (3.6)[a]
Acceleration during waking (m*g*)	31.7 (9.4)	46.1 (7.1)[f]	32.0 (3.6)[d]	32.6 (3.9)[d]	38.8 (7.1)[e]	29.3 (4.9)[c]	24.1 (2.9)[b]	24.6 (3.9)[b]	39.8 (6.5)[e]	16.9 (2.9)[a]
IG intercept	12.4 (0.7)	11.9 (0.5)[b]	12.5 (0.4)[e]	12.4 (0.4)[d]	11.5 (0.6)[a]	12.4 (0.6)[de]	12.6 (0.5)[f]	12.6 (0.6)[f]	12.2 (0.5)[c]	13.3 (0.6)[g]
IG slope	-2.1 (0.2)	-1.9 (0.1)[f]	-2.1 (0.1)[c]	-2.1 (0.1)[d]	-1.8 (0.1)[g]	-2.1 (0.2)[c]	-2.2 (0.2)[b]	-2.2 (0.2)[b]	-2.0 (0.1)[e]	-2.5 (0.2)[a]

Table S7 continued

		Cluster 1	Cluster 2	Cluster 3	Cluster 4	Cluster 5	Cluster 6	Cluster 7	Cluster 8	Cluster 9
	Total study population	RAR ++ PA ++	RAR + LIPA + Sleep -	RAR + LIPA + Sleep +	MVPA ++	RAR - Chronotype --	RAR - PA - Sleep +	RAR - PA - Sleep --	RAR - PA + Restless sleep	RAR -- PA --
N (%)	3991	501 (12.6)	587 (14.7)	626 (15.7)	379 (9.5)	333 (8.3)	705 (17.7)	366 (9.2)	234 (5.9)	260 (6.5)
Sleep										
Sleep duration (hours)	6.4 (1.0)	6.7 (0.7)^{d}	6.3 (0.8)^{b}	6.9 (0.7)^{e}	6.6 (0.8)^{cd}	6.4 (1.0)^{bc}	6.8 (0.8)^{e}	5.5 (1.0)^{a}	5.3 (0.9)^{a}	6.5 (1.1)^{bcd}
Sleep efficiency (%)	85.6 (7.7)	87.5 (4.9)^{d}	81.3 (4.9)^{c}	92.1 (2.7)^{f}	87.0 (4.5)^{cd}	86.8 (5.5)^{cd}	89.8 (3.7)^{e}	73.2 (7.7)^{a}	77.4 (9.3)^{b}	85.0 (7.2)^{c}
Mean duration of sleep bouts (min)	40.4 (17.1)	40.1 (14.1)^{c}	29.1 (5.9)^{b}	56.0 (19.2)^{d}	37.9 (11.4)^{c}	41.8 (16.4)^{c}	47.7 (16.4)^{d}	25.7 (6.9)^{a}	29.0 (10.4)^{b}	41.5 (16.8)^{c}
$TP_{ws,n}$ (%)	16.9 (5.0)	18.3 (4.1)^{ef}	14.8 (3.1)^{c}	20.7 (4.6)^{g}	17.9 (4.0)^{e}	16.8 (4.5)^{d}	18.8 (4.2)^{f}	10.7 (3.1)^{a}	12.7 (4.1)^{b}	15.2 (5.0)^{c}
Mean acceleration during sleep (m*g*)	4.01 (1.1)	3.9 (0.7)^{bc}	3.8 (0.5)^{ab}	3.8 (0.8)^{a}	3.9 (0.7)^{abc}	4.1 (1.0)^{cd}	3.9 (0.9)^{abc}	4.2 (1.1)^{d}	5.6 (2.3)^{e}	4.0 (0.9)^{abc}
Number of sleep bouts	10.8 (3.3)	10.8 (2.9)^{de}	13.5 (2.7)^{g}	8.0 (2.1)^{a}	11.1 (2.7)^{e}	10.2 (3.0)^{c}	9.4 (2.6)^{b}	13.3 (3.3)^{g}	12.0 (3.3)^{f}	10.4 (3.5)^{cd}
L_5 mean acceleration (m*g*)	4.6 (1.3)	4.3 (0.9)^{cd}	4.3 (0.7)^{cd}	4.0 (0.8)^{a}	4.3 (0.9)^{bc}	4.8 (1.4)^{e}	4.2 (0.9)^{b}	5.4 (1.3)^{f}	7.4 (2.1)^{g}	4.5 (1.1)^{de}
$TP_{sw,n}$ (%)	2.6 (1.0)	2.5 (0.8)^{c}	3.3 (0.8)^{d}	1.7 (0.5)^{a}	2.6 (0.8)^{c}	2.4 (0.8)^{c}	2.1 (0.7)^{b}	3.8 (1.0)^{e}	3.6 (1.2)^{d}	2.5 (0.9)^{c}
WASO (min)	66.1 (36.9)	57.7 (24.2)^{c}	87.9 (25.4)^{e}	35.6 (12.7)^{a}	59.4 (21.3)^{cd}	59.2 (26.9)^{cd}	47.1 (18.3)^{b}	122.0 (41.5)^{f}	96.7 (44.0)^{e}	70.0 (37.9)^{d}
Mean duration of wake bouts (min)	6.6 (2.5)	5.7 (1.3)^{bc}	7.1 (1.6)^{e}	5.1 (1.2)^{a}	5.9 (1.4)^{c}	6.5 (2.3)^{d}	5.6 (1.4)^{b}	10.3 (3.9)^{g}	8.8 (3.0)^{f}	7.4 (2.7)^{e}
Chronotype										
Sleep onset (hours of the day)	23.8 (1.0)	23.8 (0.8)^{bc}	23.6 (0.7)^{ab}	23.6 (0.7)^{a}	23.7 (0.8)^{abc}	1.4 (1.1)^{e}	23.6 (0.8)^{ab}	23.8 (1.0)^{c}	23.8 (1.0)^{abc}	0.2 (1.2)^{d}
Waking time (hours of the day)	7.4 (1.0)	7.4 (0.8)^{cd}	7.4 (0.8)^{d}	7.1 (0.8)^{b}	7.2 (0.8)^{bc}	8.8 (1.1)^{f}	7.2 (0.8)^{bc}	7.3 (1.1)^{bcd}	6.7 (1.0)^{a}	7.8 (1.2)^{e}
M_{10} start (hours of the day)	8.7 (1.2)	8.6 (1.0)^{cd}	8.5 (0.8)^{cd}	8.3 (0.9)^{a}	8.4 (1.0)^{bc}	10.4 (1.2)^{f}	8.3 (0.9)^{ab}	8.7 (1.2)^{de}	8.4 (1.2)^{abc}	9.1 (1.4)^{e}
L_5 start (hours of the day)	0.6 (1.0)	0.6 (0.8)^{bc}	0.4 (0.8)^{ab}	0.3 (0.8)^{a}	0.4 (0.8)^{a}	2.1 (1.0)^{d}	0.4 (0.8)^{ab}	0.4 (1.2)^{a}	0.2 (1.1)^{a}	0.8 (1.3)^{c}
Cosinor acrotime (hours of the day)	14.4 (1.1)	14.5 (0.9)^{e}	14.3 (0.8)^{ce}	14.1 (0.8)^{ab}	14.1 (0.9)^{ab}	16.1 (1.0)^{f}	14.0 (0.9)^{a}	14.3 (1.1)^{bcd}	14.2 (1.0)^{bcd}	14.6 (1.4)^{de}

Abbreviations: IG, intensity gradient; L_5, least active 5-hour period; LIPA, light intensity physical activity; M_{10}, most active 10-hour period; m*g*, milligravity; min, minute; MVPA, moderate to vigorous physical activity; PA, physical activity; RAR, rest-activity rhythm; SB, sedentary behaviour; $TP_{ar,d}$, transition probability from activity to rest during the day; $TP_{ra,d}$, transition probability from rest to activity during the day; $TP_{sw,n}$, transition probability from sleep to wake during the night; $TP_{ws,n}$, transition probability from wake to sleep during the night; WASO, wake after sleep onset.

*The mean scores on each metric were ranked from ^{a} to ^{h} to reflect lowest to highest mean scores across the nine circadian rhythm clusters. Tukey contrast, using the non-parametric multiple test procedure, was used to ascertain differences in ranking. The range of the ranking varied, depending on whether there were differences in ranking for the 9 clusters. When a metric has two ranks, for example the group labelled ^{ab} the implication is that it is ranked the same as the group ^{a} and the group ^{b} but the rank of group ^{a} and group ^{b} are different from each other.

Note: To improve readability, the result boxes are coloured from green (to represent good scores) to red (to represent poor scores).

Table S8. Unstandardized mean (standard deviation) scores and ranking* of each metric across the circadian rhythm clusters in the UK Biobank accelerometer sub-study.

		Cluster 1	Cluster 2	Cluster 3	Cluster 4	Cluster 5	Cluster 6	Cluster 7	Cluster 8	Cluster 9
	Total study population	RAR ++<br>PA ++	RAR +<br>PA +<br>Sleep -	RAR +<br>LIPA +<br>Sleep +	MVPA ++	RAR -<br>Chronotype --	RAR -<br>PA -<br>Sleep +	RAR -<br>PA -<br>Sleep --	RAR -<br>PA +<br>Restless sleep	RAR --<br>PA --<br>Chronotype -
N (%)	54,995	5,793 (10.5)	6,845 (12.4)	9,411 (17.1)	7,143 (13.0)	5,957 (10.8)	8,871 (16.1)	5,303 (9.6)	1,859 (3.4)	3,813 (6.9)
Rest-active rhythm										
Relative amplitude	0.85 (0.07)	0.91 (0.03)[i]	0.87 (0.04)[f]	0.88 (0.04)[g]	0.89 (0.03)[h]	0.84 (0.05)[e]	0.84 (0.05)[d]	0.80 (0.06)[c]	0.68 (0.10)[a]	0.73 (0.09)[b]
Cosinor mesor	2.44 (0.23)	2.73 (0.15)[h]	2.57 (0.14)[g]	2.53 (0.14)[f]	2.39 (0.14)[e]	2.38 (0.15)[d]	2.25 (0.15)[b]	2.34 (0.16)[c]	2.77 (0.23)[i]	2.10 (0.18)[a]
Cosinor amplitude	1.12 (0.24)	1.43 (0.16)[h]	1.25 (0.14)[g]	1.25 (0.15)[g]	1.17 (0.16)[f]	1.06 (0.16)[e]	0.99 (0.15)[d]	0.97 (0.16)[c]	0.87 (0.22)[b]	0.71 (0.17)[a]
Cosinor R^2	0.32 (0.10)	0.43 (0.08)[i]	0.38 (0.07)[g]	0.38 (0.07)[h]	0.33 (0.08)[f]	0.30 (0.08)[e]	0.28 (0.08)[d]	0.27 (0.08)[c]	0.21 (0.08)[b]	0.18 (0.08)[a]
Interdaily stability (IS)	0.57 (0.12)	0.68 (0.09)[f]	0.62 (0.09)[e]	0.62 (0.09)[e]	0.58 (0.10)[d]	0.52 (0.10)[b]	0.53 (0.10)[c]	0.53 (0.10)[c]	0.53 (0.13)[c]	0.43 (0.11)[a]
Intradaily variability (IV)	0.90 (0.23)	0.67 (0.15)[a]	0.78 (0.16)[b]	0.82 (0.16)[c]	0.89 (0.19)[d]	0.96 (0.19)[f]	1.03 (0.20)[h]	0.99 (0.20)[g]	0.92 (0.23)[e]	1.23 (0.24)[i]
Daytime activity										
SB duration (hours)	11.3 (1.9)	8.6 (1.1)[a]	10.3 (1.1)[b]	10.3 (1)[b]	11.0 (1.0)[d]	11.8 (1.1)[e]	12.5 (1.0)[f]	12.8 (1.3)[g]	10.8 (1.9)[c]	14.6 (1.4)[h]
Number of SB bouts	67.7 (13.5)	71.1 (11.9)[f]	74.8 (11.6)[g]	76.9 (11.3)[h]	58.9 (8.9)[b]	69.4 (11.6)[e]	62.6 (10.5)[c]	64.8 (11.3)[d]	76.7 (14.2)[h]	51.8 (11.8)[a]
Mean duration of SB bouts (min)	10.6 (3.7)	7.5 (1.5)[a]	8.4 (1.5)[c]	8.2 (1.4)[b]	11.5 (2.2)[f]	10.5 (2.1)[e]	12.3 (2.4)[g]	12.2 (2.6)[g]	8.7 (2.2)[d]	18.0 (6.0)[h]
$TP_{ar,d}$ (%)	22.6 (6.2)	15.6 (3.0)[a]	20.1 (3.6)[c]	21.0 (3.8)[d]	19.5 (3.6)[b]	24.4 (4.4)[e]	26.3 (4.8)[g]	25.4 (4.8)[f]	20.9 (5.0)[d]	33.4 (7.7)[h]
LIPA duration (hours)	3.9 (1.1)	4.8 (0.9)[f]	4.6 (0.8)[e]	4.8 (0.7)[f]	3.3 (0.6)[b]	3.8 (0.7)[d]	3.3 (0.6)[b]	3.4 (0.7)[c]	4.8 (1.2)[f]	2.3 (0.6)[a]
MVPA duration (min)	91.5 (51.8)	183.7 (45.9)[i]	112.5 (35.1)[g]	90.5 (29.1)[e]	117.5 (31.4)[h]	68.4 (27)[d]	51.7 (20.6)[b]	63.8 (27.0)[c]	105.7 (49.6)[f]	27.7 (17.6)[a]
Number of LIPA bouts	93.1 (20.2)	116.4 (15.0)[h]	107.2 (12.6)[g]	106.2 (12.0)[f]	85.3 (11.0)[d]	90.4 (12.8)[e]	78.5 (11.3)[b]	83.0 (12.8)[c]	107.4 (17.5)[g]	60.0 (13.4)[a]
Number of MVPA bouts	36.1 (15.8)	60.9 (12.0)[i]	45.1 (10.7)[h]	40.4 (10.4)[f]	38.4 (9.7)[e]	30.8 (9.5)[d]	23.6 (7.9)[b]	27.2 (8.9)[c]	42.6 (14.6)[g]	13.6 (7)[a]
Mean duration of LIPA bouts (min)	2.5 (0.4)	2.5 (0.4)[d]	2.6 (0.4)[f]	2.7 (0.4)[h]	2.4 (0.3)[b]	2.5 (0.4)[e]	2.5 (0.4)[e]	2.5 (0.4)[c]	2.7 (0.6)[g]	2.3 (0.3)[a]
Mean duration of MVPA bouts (min)	2.5 (0.7)	3.1 (0.7)[g]	2.5 (0.5)[f]	2.2 (0.4)[c]	3.1 (0.8)[h]	2.2 (0.5)[b]	2.2 (0.5)[b]	2.3 (0.6)[d]	2.4 (0.6)[e]	2.0 (0.7)[a]
$TP_{ra,d}$ (%)	10.7 (3.2)	14.4 (3.1)[h]	12.6 (2.4)[f]	12.8 (2.3)[g]	9.2 (1.6)[d]	10.1 (2.0)[e]	8.6 (1.6)[c]	8.8 (1.7)[b]	12.7 (3.7)[fg]	6.1 (1.5)[a]
M_{10} mean acceleration (m*g*)	49.3 (15.2)	76.0 (14.4)[i]	56.6 (8.7)[h]	51.5 (6.4)[e]	55.3 (9.9)[g]	42.8 (6.4)[d]	37.1 (5.1)[b]	40.5 (6.9)[c]	53.8 (11.8)[f]	26.9 (5.2)[a]
Acceleration during waking (m*g*)	38.6 (11.4)	59.1 (9.7)[i]	44.3 (6.1)[h]	41.1 (5.0)[e]	42.5 (6.8)[f]	34.0 (4.8)[d]	29.3 (3.8)[b]	31.1 (4.8)[c]	43.1 (9.2)[g]	21.1 (3.9)[a]
IG intercept	12.2 (0.6)	11.6 (0.6)[a]	12.1 (0.4)[b]	12.3 (0.4)[d]	11.6 (0.5)[a]	12.4 (0.4)[e]	12.5 (0.4)[f]	12.4 (0.5)[e]	12.2 (0.5)[c]	12.9 (0.6)[g]
IG slope	-2.0 (0.2)	-1.8 (0.1)[i]	-1.9 (0.1)[g]	-2.0 (0.1)[e]	-1.8 (0.1)[h]	-2.0 (0.1)[d]	-2.1 (0.1)[b]	-2.1 (0.1)[c]	-2.0 (0.1)[f]	-2.3 (0.2)[a]

Table S8. Continued

	Total study population	Cluster 1<br>RAR ++<br>PA ++	Cluster 2<br>RAR +<br>PA +<br>Sleep -	Cluster 3<br>RAR +<br>LIPA +<br>Sleep +	Cluster 4<br>MVPA ++	Cluster 5<br>RAR -<br>Chronotype --	Cluster 6<br>RAR -<br>PA -<br>Sleep +	Cluster 7<br>RAR -<br>PA -<br>Sleep --	Cluster 8<br>RAR -<br>PA +<br>Restless sleep	Cluster 9<br>RAR --<br>PA --<br>Chronotype -
N (%)	54,995	5,793 (10.5)	6,845 (12.4)	9,411 (17.1)	7,143 (13.0)	5,957 (10.8)	8,871 (16.1)	5,303 (9.6)	1,859 (3.4)	3,813 (6.9)
Sleep										
Sleep duration (hours)	6.5 (1.1)	6.8 (0.8)^{e}	6.2 (0.9)^{c}	6.8 (0.8)^{f}	7.0 (0.8)^{g}	6.5 (0.9)^{d}	6.8 (0.9)^{ef}	5.6 (1.1)^{a}	5.9 (1.2)^{b}	5.9 (1.3)^{b}
Sleep efficiency (%)	89.9 (4.9)	91.0 (3.2)^{f}	84.9 (4.0)^{b}	93.3 (2.2)^{i}	91.3 (2.8)^{g}	90.4 (2.9)^{e}	92.8 (2.4)^{h}	82.3 (5.3)^{a}	90.0 (4.4)^{d}	89.2 (4.5)^{c}
Mean duration sleep bouts (min)	52.1 (17.8)	52.8 (15.5)^{d}	37.2 (6.5)^{b}	63.5 (18.0)^{g}	54.0 (15.0)^{e}	50.0 (12.9)^{c}	62.1 (18.9)^{f}	35.3 (6.7)^{a}	51.0 (15.9)^{c}	50.2 (16.1)^{c}
TP$_{ws,n}$ (%)	19.9 (4.9)	20.8 (4.4)^{f}	17.1 (3.1)^{b}	22.2 (4.9)^{h}	20.8 (4.3)^{f}	20.0 (4.2)^{e}	21.4 (4.9)^{g}	15.4 (3.2)^{a}	19.5 (5.0)^{d}	18.4 (4.8)^{c}
Mean acceleration during sleep (*mg*)	3.6 (2.0)	3.4 (1.3)^{c}	3.3 (1.0)^{b}	3.2 (1.0)^{a}	3.3 (1.1)^{bc}	3.4 (1.1)^{bc}	3.3 (1.1)^{bc}	3.5 (1.2)^{d}	10.1 (6.3)^{f}	3.8 (1.7)^{e}
Number of sleep bouts	9.1 (2.8)	9.0 (2.3)^{e}	12.0 (2.3)^{g}	7.4 (1.9)^{a}	9.1 (2.3)^{e}	9.2 (2.2)^{e}	7.6 (2.1)^{b}	11.9 (2.8)^{f}	8.2 (2.5)^{c}	8.4 (2.6)^{d}
L$_5$ mean acceleration (*mg*)	3.9 (1.9)	3.6 (1.5)^{c}	3.9 (1.3)^{d}	3.2 (1.1)^{a}	3.3 (1.1)^{ab}	3.7 (1.3)^{c}	3.3 (1.1)^{b}	4.4 (1.6)^{f}	10.2 (4.3)^{g}	4.2 (1.6)^{e}
TP$_{sw,n}$ (%)	2.1 (0.8)	2.0 (0.6)^{d}	3.0 (0.7)^{g}	1.6 (0.5)^{a}	1.9 (0.6)^{c}	2.1 (0.6)^{e}	1.6 (0.5)^{b}	3.3 (0.8)^{h}	2.1 (0.8)^{e}	2.2 (0.8)^{f}
WASO (min)	44.1 (20.6)	40.5 (14.8)^{c}	66.5 (17.7)^{e}	30.0 (10.0)^{a}	40.6 (13.7)^{c}	42.3 (13.4)^{d}	32.4 (11.7)^{b}	72.6 (22.0)^{f}	39.5 (16.3)^{c}	42.8 (17.6)^{d}
Mean duration of wake bouts (min)	5.4 (1.5)	5.0 (1.1)^{c}	6.1 (1.3)^{g}	4.7 (1.2)^{a}	5.0 (1.1)^{c}	5.3 (1.3)^{d}	4.9 (1.3)^{b}	6.9 (1.9)^{h}	5.5 (1.7)^{e}	5.9 (1.8)^{f}
Chronotype										
Sleep onset (hours of the day)	23.9 (1.1)	23.6 (0.9)^{b}	23.7 (1.0)^{c}	23.7 (0.7)^{d}	23.5 (0.8)^{a}	1.0 (1.0)^{h}	23.6 (0.8)^{b}	0.0 (1.1)^{e}	0.4 (1.3)^{f}	0.7 (1.4)^{g}
Waking time (hours of the day)	7.1 (1.1)	7.0 (0.9)^{c}	6.9 (1.0)^{b}	7.1 (0.8)^{c}	7.2 (0.9)^{d}	8.2 (0.9)^{f}	6.9 (1.0)^{b}	6.8 (1.1)^{a}	6.9 (1.2)^{b}	7.3 (1.4)^{e}
M$_{10}$ start (hours of the day)	8.6 (1.1)	8.4 (1.0)^{bc}	8.4 (1.0)^{c}	8.4 (0.9)^{c}	8.4 (0.9)^{b}	9.7 (1.0)^{f}	8.3 (0.9)^{a}	8.4 (1.0)^{c}	8.6 (1.2)^{d}	9.1 (1.3)^{e}
L$_5$ start (hours of the day)	1.0 (1.1)	0.8 (0.9)^{b}	0.8 (1.0)^{bc}	0.9 (0.8)^{cd}	0.8 (0.9)^{b}	2.2 (0.9)^{f}	0.7 (0.9)^{a}	0.9 (1.1)^{cd}	0.9 (1.5)^{d}	1.5 (1.4)^{e}
Cosinor acrotime (hours of the day)	14.5 (1.0)	14.4 (0.9)^{c}	14.4 (0.9)^{c}	14.4 (0.7)^{d}	14.1 (0.8)^{a}	15.6 (0.9)^{f}	14.1 (0.9)^{a}	14.2 (0.9)^{b}	14.4 (1.4)^{cd}	14.8 (1.3)^{e}

Abbreviations: IG, intensity gradient; L$_5$, least active 5-hour period; LIPA, light intensity physical activity; M$_{10}$, most active 10-hour period; *mg*, milligravity; min, minute; MVPA, moderate to vigorous physical activity; PA, physical activity; RAR, rest-activity rhythm; SB, sedentary behaviour; TP$_{ar,d}$, transition probability from activity to rest during the day; TP$_{ra,d}$, transition probability from rest to activity during the day; TP$_{sw,n}$, transition probability from sleep to wake during the night; TP$_{ws,n}$, transition probability from wake to sleep during the night; WASO, wake after sleep onset.

*The mean scores on each metric were ranked from ^{a} to ^{i} to reflect lowest to highest mean scores across the nine circadian rhythm clusters. Post-hoc Tukey test with ANOVA was used to ascertain differences in ranking. The range of the ranking varied, depending on whether there were differences in ranking for the 9 clusters. When a metric has two ranks, for example the group labelled ^{ab} the implication is that it is ranked the same as the group ^{a} and the group ^{b} but the rank of group ^{a} and group ^{b} are different from each other.

Note: To improve readability, the result boxes are coloured from green (to represent good scores) to red (to represent poor scores).

Table S9. Subjective chronotype preference prevalence in the identified circadian rhythm clusters in the UK Biobank accelerometer sub-study.

	Total study population	Cluster 1	Cluster 2	Cluster 3	Cluster 4	Cluster 5	Cluster 6	Cluster 7	Cluster 8	Cluster 9
		RAR ++ PA ++	RAR + PA + Sleep -	RAR + LIPA + Sleep +	MVPA ++	RAR - Chronotype --	RAR - PA - Sleep +	RAR - PA - Sleep --	RAR - PA + Restless sleep	RAR -- PA -- Chronotype -
N (%)	46,428	4,922 (10.6)	5,855 (12.6)	7,997 (17.2)	6,036 (13.0)	4,960 (10.7)	7,471 (16.1)	4,446 (9.6)	1,594 (3.4)	3,147 (6.8)
Chronotype preference, N (%)[a]										
Definitely a morning person	12,645 (27.2)	1653 (33.6)	1816 (31.0)	2197 (27.4)	1855 (30.7)	573 (11.6)	2215 (29.6)	1298 (29.2)	423 (26.5)	615 (19.5)
More a morning than evening person	18,055 (38.9)	2001 (40.7)	2378 (40.6)	3343 (41.8)	2540 (42.1)	1335 (26.9)	3086 (41.3)	1782 (40.1)	584 (36.6)	1006 (32.0)
More an evening than a morning person	11,940 (25.7)	1020 (20.7)	1358 (23.2)	1977 (24.7)	1358 (22.4)	1964 (39.6)	1748 (23.4)	1054 (23.7)	441 (27.7)	1020 (32.4)
Definitely an evening person	3,788 (8.2)	248 (5.0)	303 (5.2)	480 (6.0)	283 (4.7)	1088 (21.9)	422 (5.6)	312 (7.0)	146 (9.2)	506 (16.1)

Abbreviations: LIPA, light intensity physical activity; MVPA, moderate to vigorous physical activity; PA, physical activity; RAR, rest-active rhythm.
5,079 NAs for the variable chronotype preference.
[a]Percentage of the category for the column.

Table S10. Distribution of shift work in the identified clusters in the UK Biobank accelerometer sub-study.

	Total study population	Cluster 1<br>RAR ++<br>PA ++	Cluster 2<br>RAR +<br>PA +<br>Sleep -	Cluster 3<br>RAR +<br>LIPA +<br>Sleep +	Cluster 4<br>MVPA ++	Cluster 5<br>RAR -<br>Chronotype --	Cluster 6<br>RAR -<br>PA -<br>Sleep +	Cluster 7<br>RAR -<br>PA -<br>Sleep --	Cluster 8<br>RAR -<br>PA +<br>Restless sleep	Cluster 9<br>RAR --<br>PA --<br>Chronotype -
Job involving shift work (%)										
No	30,026 (87.1)	3,144 (86.3)	3,578 (85.8)	5,055 (88.1)	4,268 (87.4)	3,292 (89.1)	5,025 (87.4)	2,813 (85.7)	914 (87.0)	1,937 (85.5)
Yes	4,441 (12.9)	498 (13.7)	593 (14.2)	680 (11.9)	613 (12.6)	403 (10.9)	722 (12.6)	468 (14.3)	136 (13.0)	328 (14.5)

Abbreviations: LIPA, light intensity physical activity; MVPA, moderate to vigorous physical activity; PA, physical activity; RAR, rest-active rhythm.

20,528 NAs for the variable shift work.

Figure S1. Flow-chart of sample selection in the Whitehall II accelerometer sub-study.

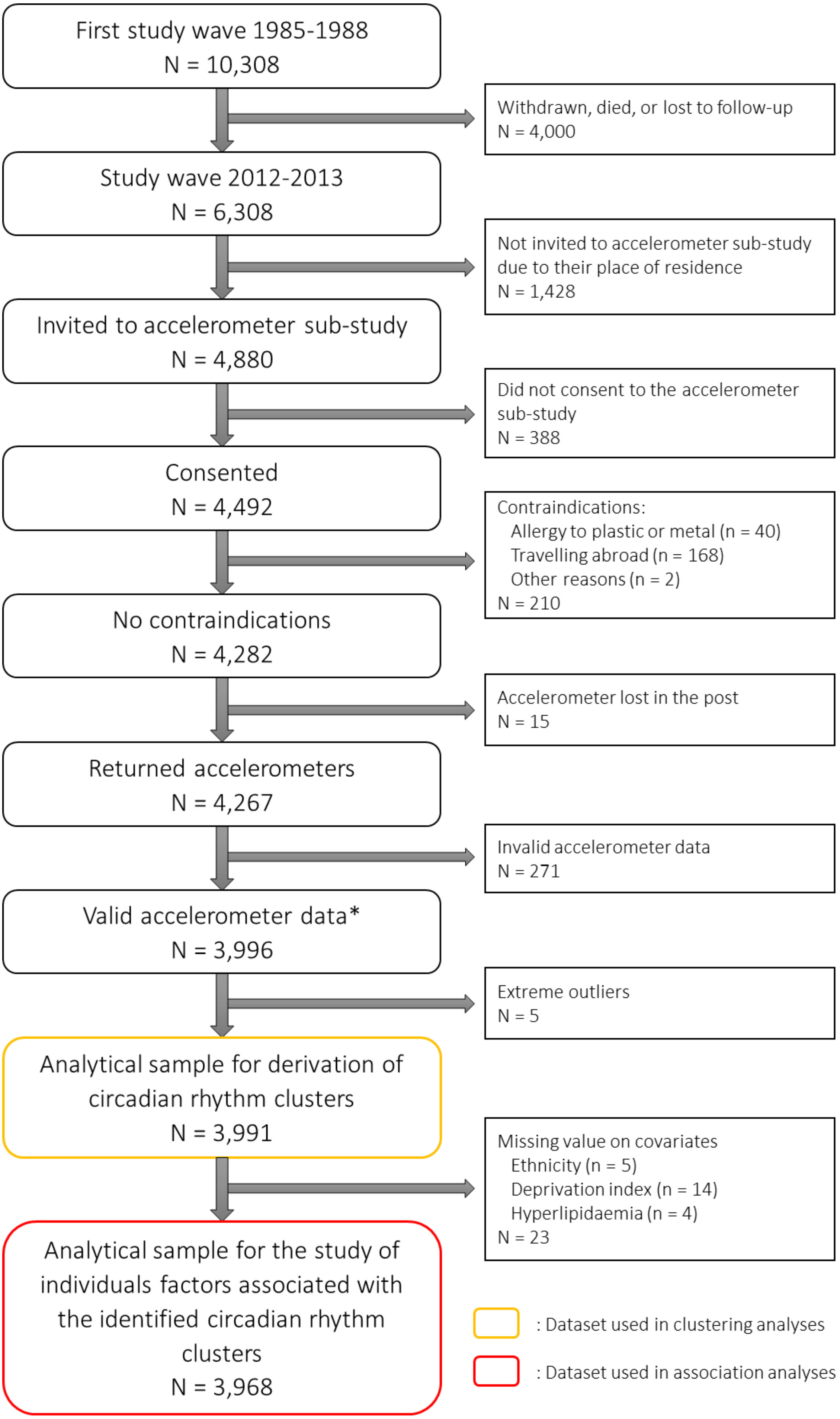

Abbreviations: ADL, activity of daily living.

*Corresponds to 5 valid days defined as both wear times during waking period and the following sleep period ≥2/3 of the respective periods.

Figure S2. Flow-chart of sample selection in the UK Biobank accelerometer sub-study.

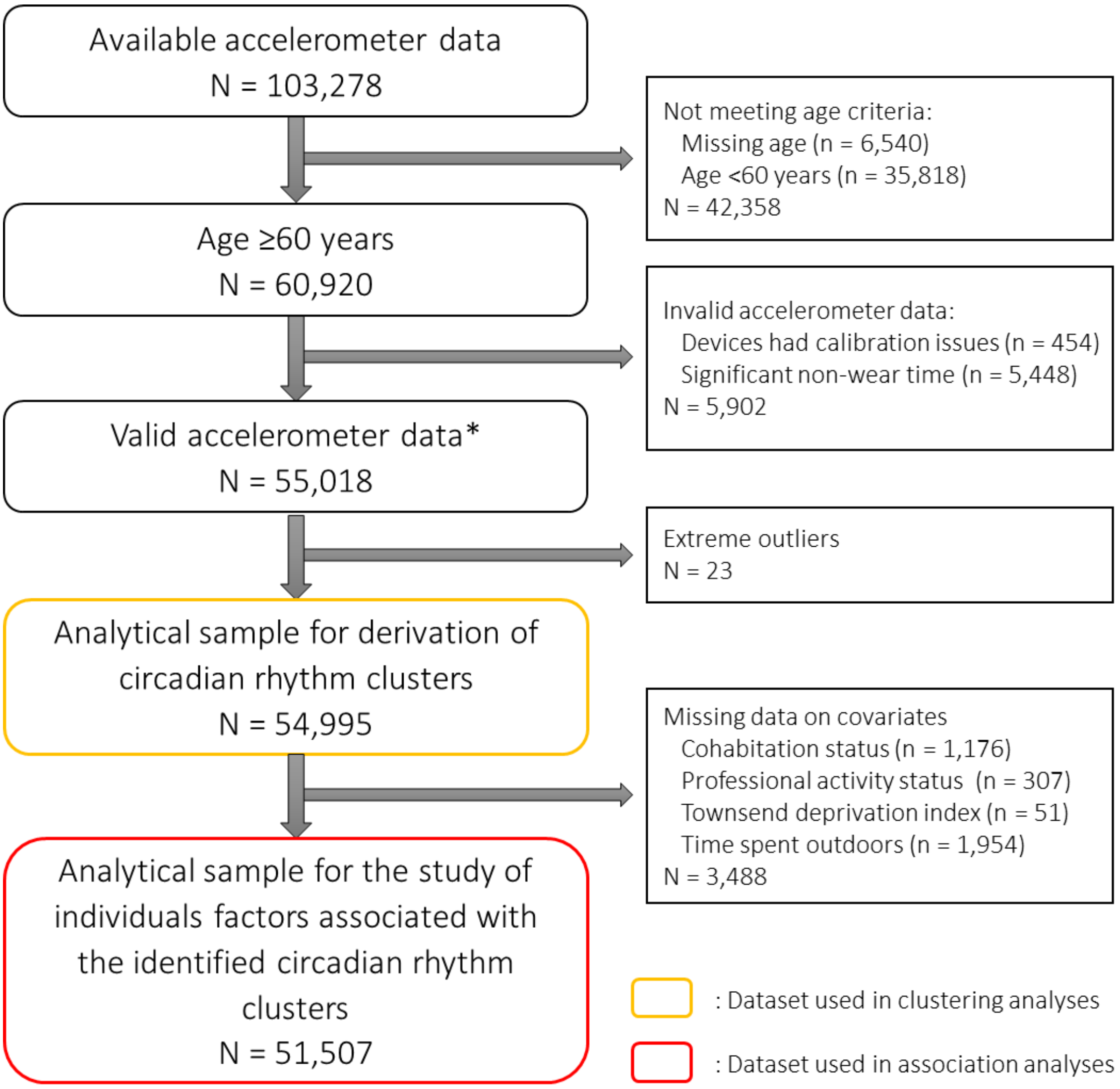

*Corresponds to 5 valid days defined as both wear times during sleep period and the following waking period ≥2/3 of the respective periods.

Figure S3. Work-flow of the statistical analysis for clusters identification

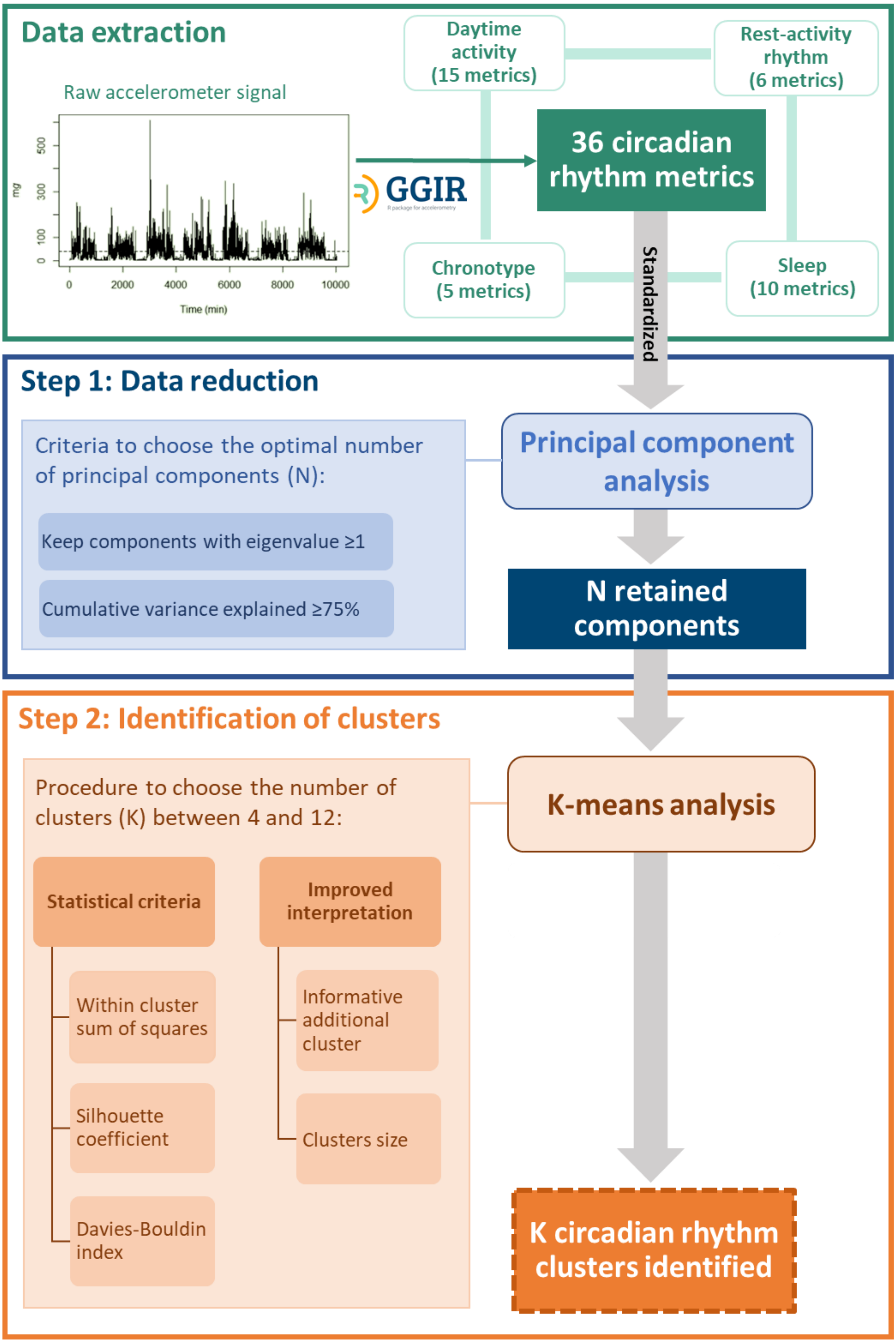

Figure S4. Statistical criteria results for the clustering solutions with K (number of clusters) between 4 and 12 in Whitehall II (n=3,991).

Statistical criteria	Results	Conclusion
Within cluster sum of squares (WCSS)<br>We are in search of an elbow between K=4 to K=12	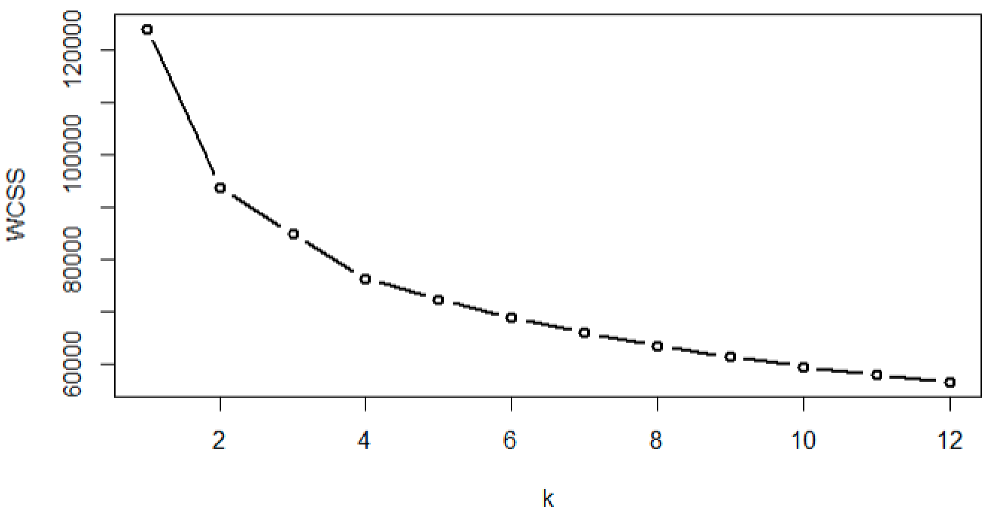	No clear solution
Silhouette coefficient<br>We search for the highest value	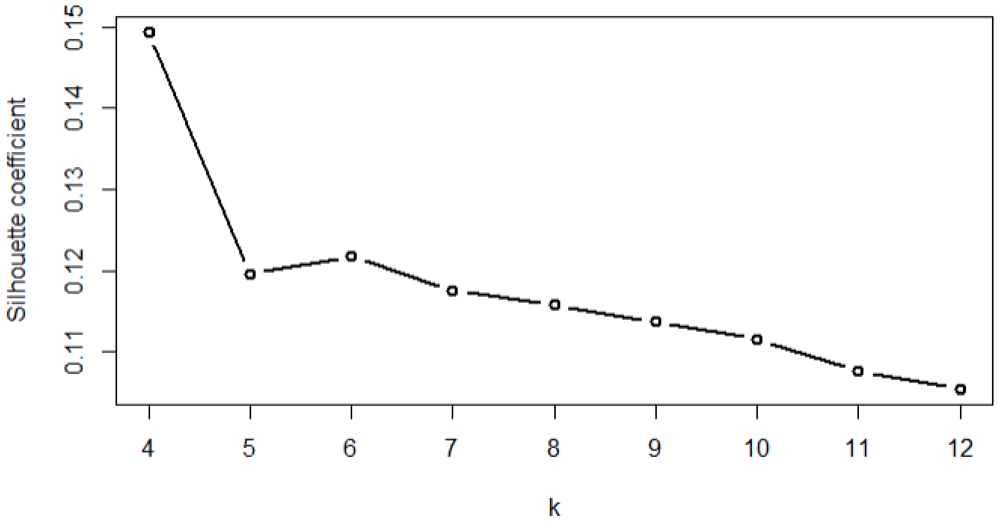	Best solution is K=4
Davies-Bouldin index<br>We search for the lowest value	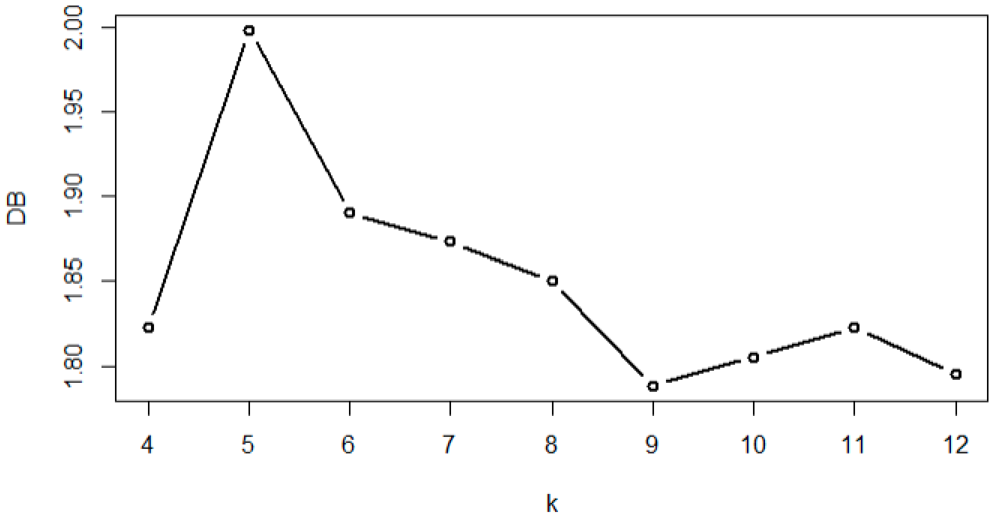	Best solution is K=9
Overall	No clear solution although solutions do not strongly differ in terms of statistical performance for the different statistical tool, leading to further insight to interpretability of the clusters.	

Source data are provided as a Source Data file.

Figure S5. Statistical criteria results for the clustering solutions with K (number of clusters) between 4 and 12 in UK Biobank (n=54.995).

<table>
<tr><td>Statistical criteria</td><td>Results</td><td>Conclusion</td></tr>
<tr><td>Within cluster sum of squares (WCSS)
We are in search of an elbow between K=4 to K=12</td><td>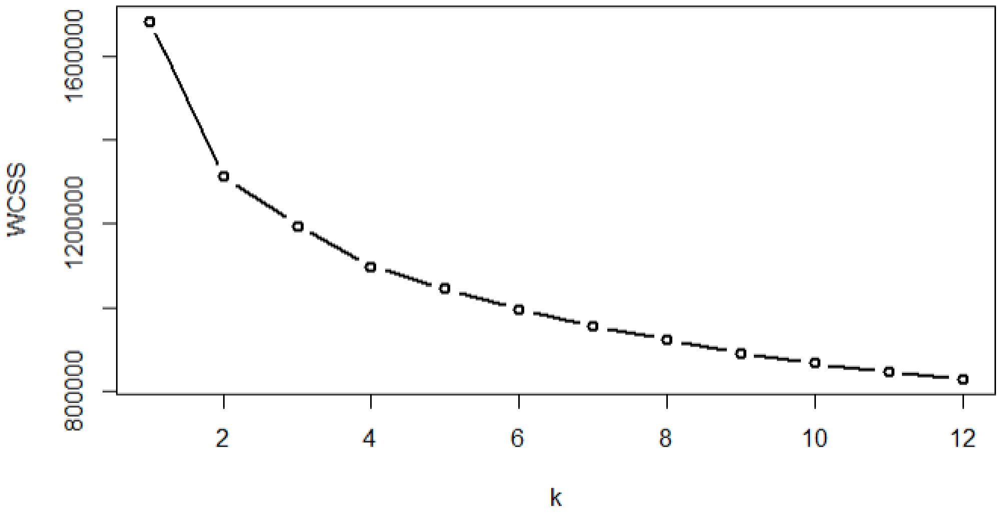
</td><td>No clear solution</td></tr>
<tr><td>Silhouette coefficient
We search for the highest value</td><td>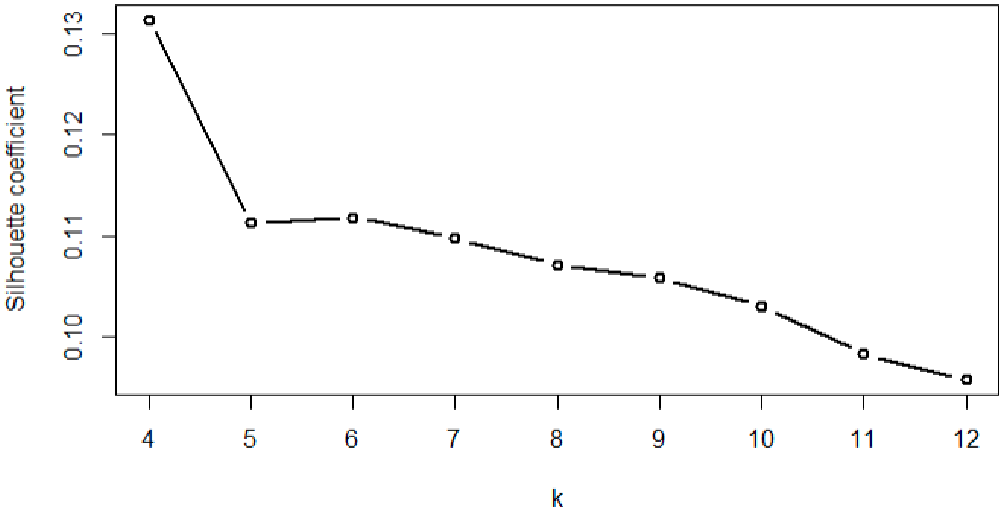
</td><td>Best solution is K=4</td></tr>
<tr><td>Davies-Bouldin index
We search for the lowest value</td><td>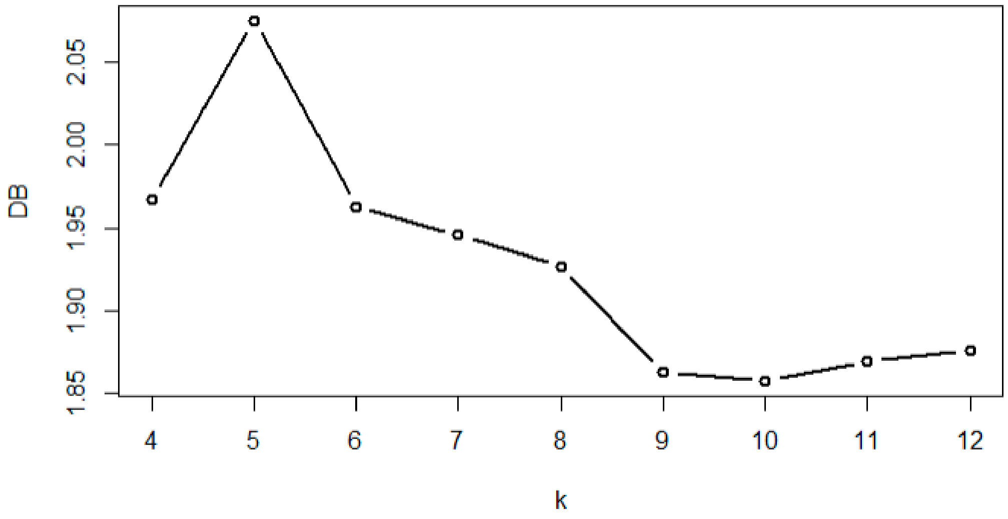
</td><td>Best solution is K=10</td></tr>
<tr><td>Overall</td><td colspan="2">No clear solution although solutions do not strongly differ in terms of statistical performance for the different statistical tool, leading to further insight to interpretability of the clusters.</td></tr>
</table>

Source data are provided as a Source Data file.

Figure S6. Illustration of acceleration signals for typical profiles for the nine clusters identified in the Whitehall II accelerometer sub-study.

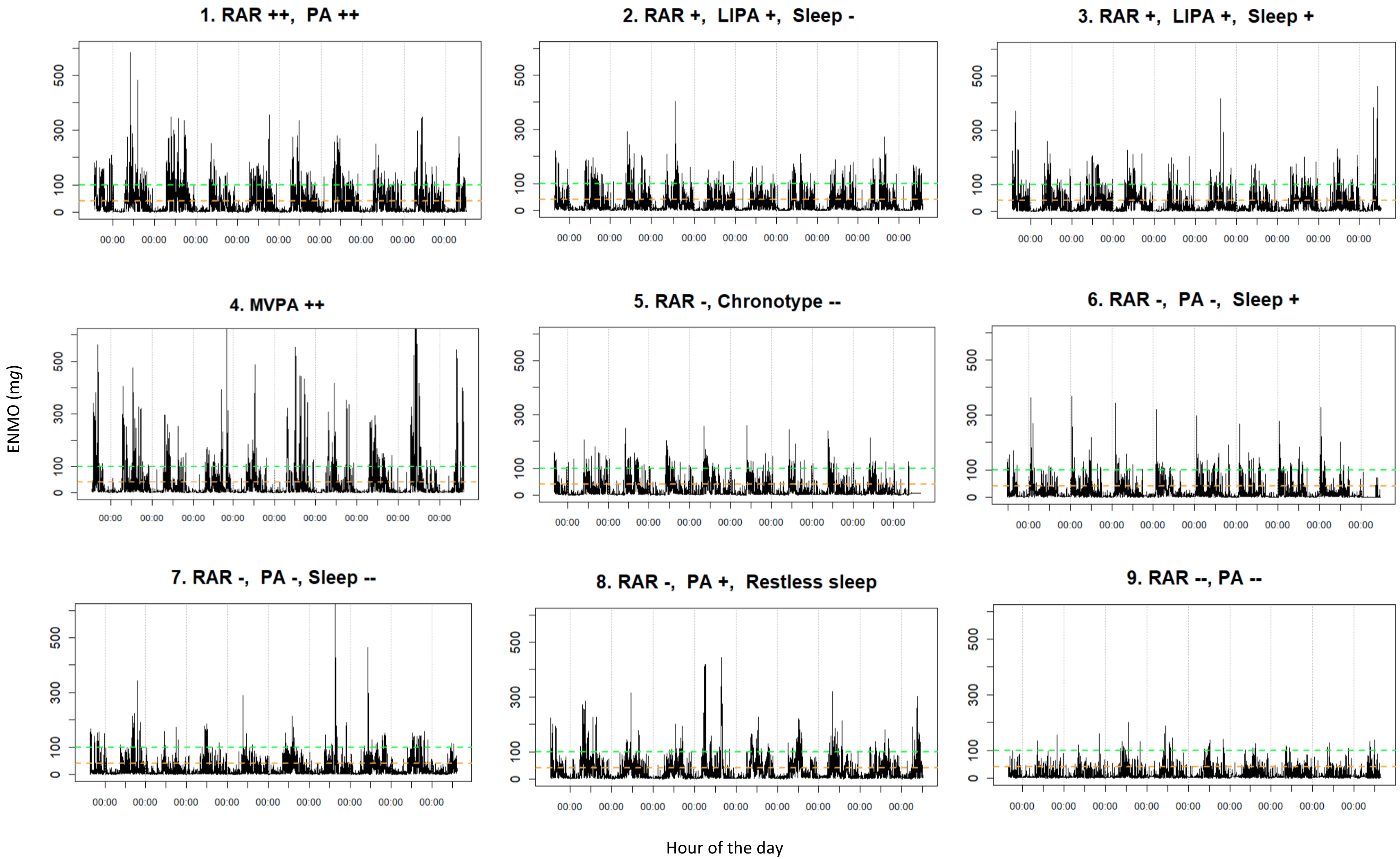

Abbreviations: ENMO, Euclidean norm minus one; LIPA, light intensity physical activity; m*g*, milligravity; MVPA, moderate to vigorous physical activity; PA, physical activity; RAR, rest-activity rhythm.
Acceleration signal of the medoid (median) individual for the nine clusters over the observation period. Horizontal orange dotted line corresponds to 40 m*g*, threshold of transition between sedentary behaviour and LIPA. Horizontal green dotted line corresponds to 100 m*g*, threshold of transition between LIPA and MVPA. Vertical grey dotted lines correspond to midnight time for each day.

Figure S7. Illustration of acceleration signals for typical profiles for the nine clusters identified in the UK Biobank accelerometer sub-study.

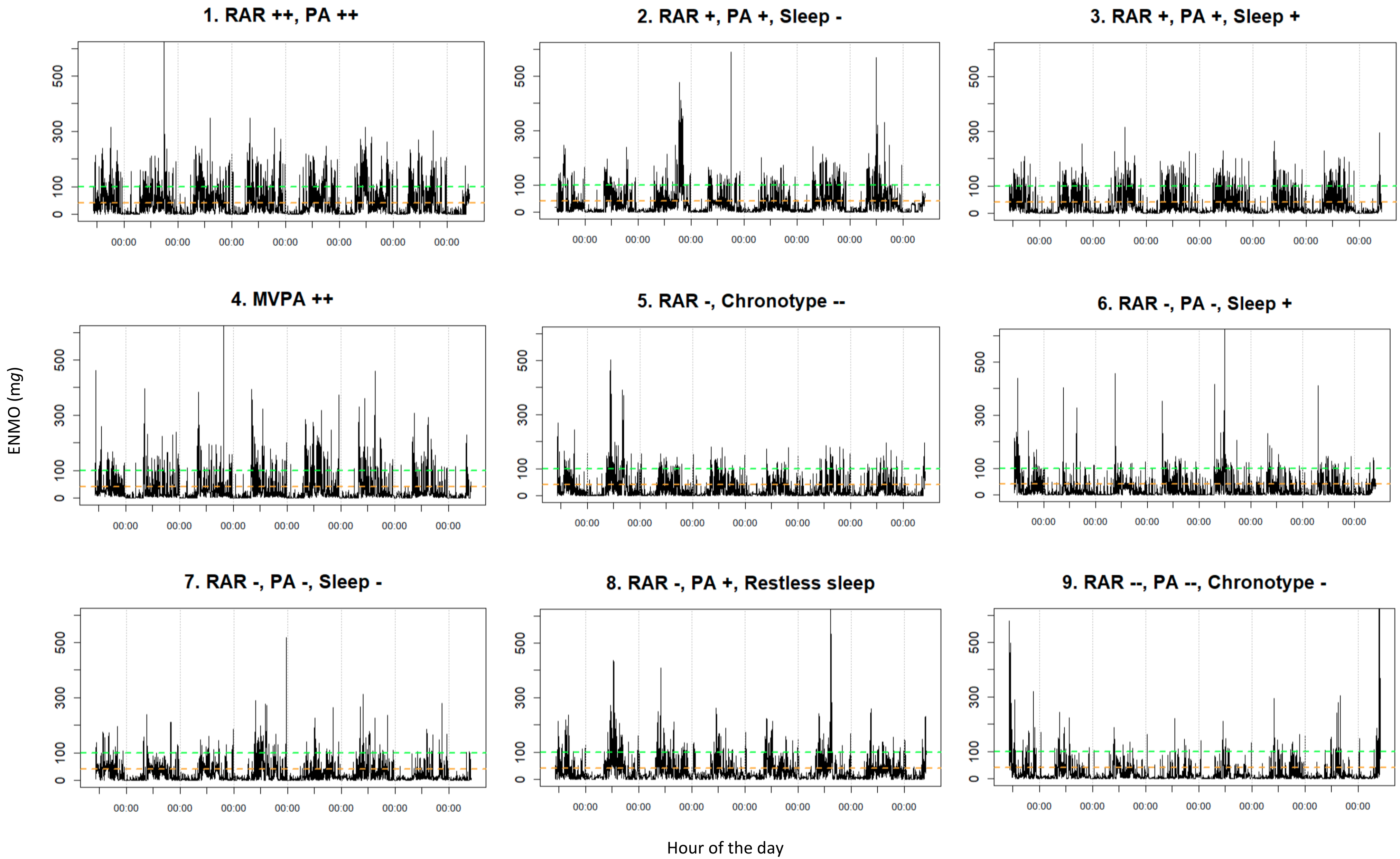

Abbreviations: ENMO, Euclidean norm minus one; LIPA, light intensity physical activity; m*g*, milligravity; MVPA, moderate to vigorous physical activity; PA, physical activity; RAR, rest-activity rhythm.
Acceleration signal of the medoid (median) individual for the nine clusters over the observation period. Horizontal orange dotted line corresponds to 40 m*g*, threshold of transition between sedentary behaviour and LIPA. Horizontal green dotted line corresponds to 100 m*g*, threshold of transition between LIPA and MVPA. Vertical grey dotted lines correspond to midnight time for each day.
