# Supplementary material for: Circadian rhythm profiles derived from accelerometer measures of the sleep-wake cycle in two cohort studies
Source: Nat Commun. 2025 Dec 13;16:11357. doi: 10.1038/s41467-025-66407-2 (PMC12727707; doi:10.1038/s41467-025-66407-2)
Supplement: Supplementary file 3 — Reporting Summary [file 41467_2025_66407_MOESM3_ESM.pdf]

nature portfolio

Corresponding author(s): Séverine Sabia

Last updated by author(s): Oct 21, 2025

Reporting Summary

Nature Portfolio wishes to improve the reproducibility of the work that we publish. This form provides structure for consistency and transparency in reporting. For further information on Nature Portfolio policies, see our Editorial Policies and the Editorial Policy Checklist.

Statistics

For all statistical analyses, confirm that the following items are present in the figure legend, table legend, main text, or Methods section.

n/a	Confirmed	
☐	☒	The exact sample size (*n*) for each experimental group/condition, given as a discrete number and unit of measurement
☐	☒	A statement on whether measurements were taken from distinct samples or whether the same sample was measured repeatedly
☐	☒	The statistical test(s) used AND whether they are one- or two-sided<br>*Only common tests should be described solely by name; describe more complex techniques in the Methods section.*
☐	☒	A description of all covariates tested
☐	☒	A description of any assumptions or corrections, such as tests of normality and adjustment for multiple comparisons
☐	☒	A full description of the statistical parameters including central tendency (e.g. means) or other basic estimates (e.g. regression coefficient) AND variation (e.g. standard deviation) or associated estimates of uncertainty (e.g. confidence intervals)
☐	☒	For null hypothesis testing, the test statistic (e.g. *F*, *t*, *r*) with confidence intervals, effect sizes, degrees of freedom and *P* value noted<br>*Give P values as exact values whenever suitable.*
☒	☐	For Bayesian analysis, information on the choice of priors and Markov chain Monte Carlo settings
☐	☒	For hierarchical and complex designs, identification of the appropriate level for tests and full reporting of outcomes
☐	☒	Estimates of effect sizes (e.g. Cohen's *d*, Pearson's *r*), indicating how they were calculated

Our web collection on statistics for biologists contains articles on many of the points above.

Software and code

Policy information about availability of computer code

Data collection: No software was used to collect data.

Data analysis: We used GGIR version 3.1-7 to extract the data from raw data to metrics used. And for the analyses we used different functions in R version 4.2.2. All of these can be found at: https://github.com/samvidil/Clusters-of-circadian-rhythm-article and in Zenodo at: https://doi.org/10.5281/zenodo.17417859

For manuscripts utilizing custom algorithms or software that are central to the research but not yet described in published literature, software must be made available to editors and reviewers. We strongly encourage code deposition in a community repository (e.g. GitHub). See the Nature Portfolio guidelines for submitting code & software for further information.

Data

Policy information about availability of data

All manuscripts must include a data availability statement. This statement should provide the following information, where applicable:

- Accession codes, unique identifiers, or web links for publicly available datasets
- A description of any restrictions on data availability
- For clinical datasets or third party data, please ensure that the statement adheres to our policy

Data cannot be made publicly available because of ethics and IRB restrictions. However, a data sharing portal allows access to data to undertake analyses within the secure portal in WII https://portal.dementiasplatform.uk/. The UK Biobank data are available through a procedure described at https://www.ukbiobank.ac.uk/

enable-your-research.
Source data are provided with this paper.

Research involving human participants, their data, or biological material

Policy information about studies with human participants or human data. See also policy information about sex, gender (identity/presentation), and sexual orientation and race, ethnicity and racism.

Reporting on sex and gender	In Whitehall II sex was defined based on the British Civil service registry. In UK Biobank sex was defined based on the combination of NHS central registry and self report from the participant.<br>All the results are applicable to both sexes.
Reporting on race, ethnicity, or other socially relevant groupings	We used self-reported ethnicity in both cohorts.<br>This variable was defined as "white" or "non-white".
Population characteristics	In Whitehall II, mean age = 69.4 years, standard deviation=5.7years, 26% were women, 8% were non white ethnicity, 42% were at "level lower secondary school or less" and 25% lived alone.<br><br>In UK Biobank, mean age = 67.5 years, standard deviation=4.2years, 54% were women, 2% were non white ethnicity, 11% were at "level lower secondary school or less" and 18% lived alone.<br>These informations are available in Tables 1 and 2.
Recruitment	All persons aged 35 to 55 years working in 20 London based departments of the British civil service in 1985-1988 were invited to participate by letter and 73% agreed. An accelerometer measure was added to the 2012-2013 wave of data collection for the 4,880 participants seen at the London clinic and those living in the South-Eastern regions of England who underwent clinical examination at home<br><br>9,238,453 individuals registered with the UK's National Health Service who were aged 40–69 years and lived within approximately 25 miles (40 km) of one of 22 assessment centers located throughout England, Wales, and Scotland were invited between 2006 and 2010. Among them, 503317 agreed (5.45%). 236,519 UK Biobank participants were approached between 2013 and 2015 to wear an accelerometer, of whom 106,053 agreed (44.8%). For these analyses we retained those aged 60 or more.
Ethics oversight	University College London ethics committee for Whitehall II and National Information Governance Board for Health.<br>Social Care and the National Health Service North West Centre for Research Ethics Committee for UK Biobank.

Note that full information on the approval of the study protocol must also be provided in the manuscript.

Field-specific reporting

Please select the one below that is the best fit for your research. If you are not sure, read the appropriate sections before making your selection.

☒ Life sciences ☐ Behavioural & social sciences ☐ Ecological, evolutionary & environmental sciences

For a reference copy of the document with all sections, see nature.com/documents/nr-reporting-summary-flat.pdf

Life sciences study design

All studies must disclose on these points even when the disclosure is negative.

Sample size	Sample size were not decided specifically for the paper as data come from two ongoing cohort established between 2012-2013 for Whitehall II and 2013-2015 for UK Biobank
Data exclusions	Reasons for exclusion in the analyses include: not being in the target age at accelerometer wearing (younger than 60 years), invalid accelerometer data, extreme outliers, and missing values on covariates.<br><br>In Whitehall II, data on accelerometer-assessed circadian rhythm metrics collected in 2012-2013 were available for 4267 participants. Data were excluded due to invalid accelerometer data (n=271), extreme outliers (n=5), leading to a total of 3991 participants included in the clustering analysis, and missing covariates (n=23) leading to a total of 3968 participants in the association analyses.<br><br>In UK Biobank, data on accelerometer-assessed circadian rhythm metrics collected in 2013-2015 were available on 103278 participants. Data were excluded due to not meeting age criteria (<60 years) (n=42358), invalid accelerometer data (n=5902), extreme outliers (n=23), leading to a total of 54995 participants included in the clustering analysis, and missing covariates (n=3488) leading to a total of 51507 participants in the association analyses.
Replication	We ensured robustness of the findings by conducting the analyses in two separate cohorts leading to similar results.
Randomization	A large set of covariates was used to control for potential confounding factors: age, sex, ethnicity, education, marital status, deprivation index, season when accelerometer was worn, alcohol consumption, smoking status, fruit and vegetable consumption, light exposure, BMI, hypertension, diabetes, hyperlipidemia, prevalence of chronic disease (among coronary heart disease, stroke, Parkinson’s disease, chronic obstructive pulmonary disease, heart failure, depression, other mental disorders, cancer, liver disease, and arthritis), and central nervous

	system medication.<br>In Whitehall II, we also used in supplementary, analyses GHQ depression, and ADL limitations.
Blinding	Circadian rhythm metrics were assessed with accelerometer which allows objective measure. All the factors were either assessed by questionnaire, clinical examination, or data from electronic health records. Statistical analysis conducted by the first author of the paper was not blinded as information on both circadian rhythm and different factors was needed to conduct the analysis.

Reporting for specific materials, systems and methods

We require information from authors about some types of materials, experimental systems and methods used in many studies. Here, indicate whether each material, system or method listed is relevant to your study. If you are not sure if a list item applies to your research, read the appropriate section before selecting a response.

Materials & experimental systems

n/a	Involved in the study
☒	☐ Antibodies
☒	☐ Eukaryotic cell lines
☒	☐ Palaeontology and archaeology
☒	☐ Animals and other organisms
☒	☐ Clinical data
☒	☐ Dual use research of concern
☒	☐ Plants

Methods

n/a	Involved in the study
☒	☐ ChIP-seq
☒	☐ Flow cytometry
☒	☐ MRI-based neuroimaging

Plants

Seed stocks	*Report on the source of all seed stocks or other plant material used. If applicable, state the seed stock centre and catalogue number. If plant specimens were collected from the field, describe the collection location, date and sampling procedures.*
Novel plant genotypes	*Describe the methods by which all novel plant genotypes were produced. This includes those generated by transgenic approaches, gene editing, chemical/radiation-based mutagenesis and hybridization. For transgenic lines, describe the transformation method, the number of independent lines analyzed and the generation upon which experiments were performed. For gene-edited lines, describe the editor used, the endogenous sequence targeted for editing, the targeting guide RNA sequence (if applicable) and how the editor was applied.*
Authentication	*Describe any authentication procedures for each seed stock used or novel genotype generated. Describe any experiments used to assess the effect of a mutation and, where applicable, how potential secondary effects (e.g. second site T-DNA insertions, mosiacism, off-target gene editing) were examined.*
